# Clinical outcomes of children with acute asthma and pneumonia in Mulago hospital, Uganda: a prospective study

**DOI:** 10.1186/s12887-014-0285-4

**Published:** 2014-11-28

**Authors:** Rebecca Nantanda, Marianne S Ostergaard, Grace Ndeezi, James K Tumwine

**Affiliations:** Child Health and Development Centre, Makerere University College of Health Sciences, Kampala, Uganda; The Research Unit for General Practice and Section of General Practice, Department of Public Health, University of Copenhagen, Copenhagen, Denmark; Department of Paediatrics and Child Health, Makerere University College of Health Sciences, Kampala, Uganda

**Keywords:** Asthma, Pneumonia, ‘Under-fives’, Duration of hospitalization, Mortality

## Abstract

**Background:**

Little attention has been paid to asthma in ‘under-fives’ in Sub-Saharan Africa. In ‘under-fives’, acute asthma and pneumonia have similar clinical presentation and most children with acute respiratory symptoms are diagnosed with pneumonia according to the WHO criteria. The mortality associated with acute respiratory diseases in Uganda is high but improving, dropping from 24% in 2004 to 11.9% in 2012. We describe the immediate clinical outcomes of children with acute asthma and pneumonia and document the factors associated with prolonged hospitalization and mortality.

**Methods:**

We enrolled 614 children aged 2 to 59 months with acute respiratory symptoms presenting at the emergency paediatric unit of Mulago hospital. Clinical histories, physical examination, blood and radiological tests were done. Children with asthma and bronchiolitis were collectively referred to as ‘Asthma syndrome’. Hospitalized children were monitored every 12 hours for a maximum of 7 days. Survival analysis was done to compare outcome of children with asthma and pneumonia. Cox regression analysis was done to determine factors associated with prolonged hospitalization and mortality.

**Results:**

Overall mortality was 3.6%. The highest case fatality was due to *pneumocystis jirovecii* pneumonia (2/4) and pulmonary tuberculosis (2/7). None of the children with asthma syndrome died. Children with ‘asthma syndrome’ had a significantly shorter hospital stay compared to those with pneumonia (p<0.001). Factors independently associated with mortality included hypoxemia (HR = 10.7, 95% CI 1.4- 81.1) and severe malnutrition (HR = 5.7, 95% CI 2.1- 15.8). Factors independently associated with prolonged hospitalization among children with asthma syndrome included age less than 12 months (RR = 1.2, 95% CI 1.0-1.4), hypoxemia (RR = 1.4, 95% CI 1.2-1.7), and severe malnutrition (RR = 1.5 95% CI 1.3-1.8). Similar factors were associated with long duration of hospital stay among children with pneumonia.

**Conclusion:**

This study identified a sharp decline in acute respiratory mortality compared to the previous studies in Mulago hospital. This may be related to focus on and treatment of asthma in this study, and will be analysed in a later study. Bacterial pneumonia is still associated with high case fatality. Hypoxemia, severe malnutrition, and being an infant were associated with poor prognosis among children with acute asthma and pneumonia and need to be addressed in the management protocols.

**Electronic supplementary material:**

The online version of this article (doi:10.1186/s12887-014-0285-4) contains supplementary material, which is available to authorized users.

## Background

Pneumonia and acute asthma are different disease entities with similar clinical presentation among young children [1]. The diagnostic gold standards for pneumonia and acute asthma in young children are quite sensitive but very unspecific and may be difficult to apply in low-income settings like Uganda [2,3]. Consequently, using the current guidelines for diagnosis of pneumonia and acute asthma creates challenges in differentiating the two conditions. Hence, some children with acute asthma are misdiagnosed as pneumonia [4,5]. In a recent study in Mulago hospital our team showed that, of the 614 children who presented with acute respiratory symptoms, 41.2% were diagnosed with ‘asthma syndrome’ *post-hoc*, by a panel of paediatricians and pulmonologists. Of these, 95% had prescriptions for antibiotics although only 19.8% had combined asthma and bacterial pneumonia [6]. In such circumstances, the outcomes of children with acute asthma are attributed to pneumonia. This may impact on management protocols for children with asthma, such as referral for chronic care, health education on prevention of exacerbations and home management of asthma attacks.

Acute asthma is a common cause of emergency hospital visits in low and medium income countries [7-9] Studies in developed countries show that outcomes of children with acute asthma are influenced by age of the child, peripheral oxygen saturation on admission and adherence to asthma therapy [10-12]. Presence of co-morbidities such as pneumonia and acute upper respiratory tract infections (URTI) also affects outcomes [13,14]. Hitherto, no studies in Uganda have described factors associated with outcomes of children with acute asthma.

Pneumonia has been considered the major cause of morbidity and mortality among children less than five years. Diagnosis is usually based on presence of cough and/or difficult breathing, fast breathing with/without chest in drawing [15]. The previous studies among ‘under-fives’ in Mulago hospital [16-18], using the above WHO definition for pneumonia, documented high pneumonia case fatality ratios ranging from 24% in 2004 to 11.9% in 2012 [16-18]. However, several studies have indicated that this definition is very non-specific and includes other diseases which closely mimic pneumonia such as acute asthma and bronchiolitis [4,19,20]. It has been hypothesized that undiagnosed and hence untreated asthma may be contributing to respiratory mortality among children less than five years [1]. In some of the studies among children with acute lower respiratory symptoms, mortality was more likely in those with prolonged cough, recurrent respiratory symptoms and, fast breathing and hypoxia but without fever [21]. Even though children with pneumonia may present without fever [22], the presence of the recurrent symptoms in these children may imply a diagnosis of asthma rather than pneumonia. Similarly, in low-income countries, Respiratory Syncytial Virus (RSV) has been associated with treatment failure and mortality [23]. However, viral infections are generally mild and self-limiting. Hence, the RSV-associated treatment failure and mortality may in fact be due to untreated underlying asthma [23,24].

The objective of our study was to compare the immediate clinical outcome of children with acute asthma, pneumonia or combined asthma and pneumonia. This is the first study among children less than five years with acute respiratory symptoms in Mulago hospital Uganda, with a focus on outcomes of acute asthma. We hypothesized that children with a combination of acute asthma and pneumonia would have longer duration of hospitalization compared to those with acute asthma or pneumonia alone. We also sought to describe the factors associated with prolonged hospitalization and mortality among children with acute asthma and pneumonia. The findings might help in identification of children at high risk of adverse outcomes and inform management protocols for children with acute respiratory illnesses.

## Methods

### Study design and setting

We conducted a prospective study of children with acute respiratory symptoms aged 2 to 59 months presenting at the emergency paediatric unit of Mulago hospital Kampala between August 2011 and June 2012. Mulago hospital is a national referral and teaching hospital for Makerere University. The unit comprises of the paediatric intensive care unit (PICU) and high dependency ward. Upon stabilisation, the children are transferred to other wards for further care. The unit attends to children aged 1 day up to 12 years with severe illnesses. The average daily attendance is 80 children, 75% of whom are aged 2 to 59 months. An estimated 25% of the children present with cough and difficulty in breathing. The hospital was selected as the study site because of its ability to handle laboratory and radiological investigations that were used to diagnose asthma and pneumonia, facilities that are not readily available in rural Ugandan hospitals.

### Ethical consideration

The study was approved by the Higher Degrees, Ethics and Research Committee (HDREC) at Makerere University College of Health Sciences and the Uganda National Council of Science and Technology (UNCST). Informed written consent was obtained from the caretakers of the participants. This study conforms to the STROBE guidelines for reporting observational studies [25] as described in Additional file 1.

### Recruitment, management and follow up of the participants

All children attending the paediatric emergency unit at Mulago hospital were screened and those aged 2–59 months with cough and/or difficult breathing and fast breathing, with/without chest in-drawing were consecutively enrolled after obtaining informed written consent from the caretakers. We excluded children with known heart conditions and those with heart failure secondary to severe anaemia. All participants were triaged and those with ‘severe classification’ according to the WHO guidelines [26] were given urgent care before proceeding with the consent process. A questionnaire focusing on the clinical history of the child was administered by a nurse. The doctor performed the clinical examination. For all participants, we measured the peripheral oxygen saturation (SaO_2_) in room air. Children with SaO_2_ less than 92% were given oxygen by mask, nasal catheter or prongs. Nutritional assessment was done according to WHO guidelines on management of children with severe malnutrition [26]. The children who had chest in-drawing were admitted and followed up to determine outcomes. The details of the laboratory methods have been described elsewhere [27]. Briefly; blood culture, total and differential white cell counts, and serum C-reactive protein (CRP) titres were done. In addition, a peripheral blood smear for malaria parasites, HIV testing, a nasopharyngeal swab for identification of Respiratory Syncytial Virus (RSV) and chest x-ray were done. Additional consent for HIV testing was sought from the caretakers. The results of the tests were used to aid the diagnosis of either asthma or pneumonia, guided by the study diagnostic categories as previously described [6].

Children with wheezing were nebulised with salbutamol solution using an ultrasonic nebulizer and the response noted. Children with wheezing and chest indrawing were given oral Predinisolone and for those who were unable to take the oral Predinisolone, intravenous Hydrocortisone was given. Children who had pneumonia were treated with antibiotics [28]. Hospitalized children were followed up every 12 hours until discharge, death or for a maximum of seven [7] days, whichever came first. For children who were hospitalized for more than 7 days, the date of discharge was recorded. Measurements included axillary temperature, arterial peripheral oxygen saturation, respiratory rate, presence of chest in drawing, wheezing (audible and auscultatory), and ability to feed. To determine the oxygen saturation, we disconnected the oxygen from the patients for 5 minutes before taking the final measurement. We counted the respiratory rate for 60 seconds, using a timer and in accordance with the WHO Integrated Management of Childhood Illnesses (IMCI) guidelines [2]. This was an observation study and there was no active intervention from the research team except to give bronchodilators to the children who were wheezing because this was part of the study protocol. The test results and any clinical features that needed attention of the attending doctors were duly communicated. The decision to discharge the patients was at the discretion of the attending doctors.

### Variables

The primary outcome measures were duration of hospitalization and mortality. The secondary outcomes were: time to normalization of the respiratory rate and time taken for oxygen saturation to normalize. Furthermore, we described the factors associated with prolonged hospitalization and mortality, and these included; age of the child, hypoxia, nutritional status, exclusive breastfeeding, level of education of caretaker, HIV status and gender.

### Definitions

The study definitions were formulated based on current international guidelines and consultation with experts.

***Asthma:*** The definition of asthma was based on modified Global Initiative for Asthma (GINA) guidelines for asthma management and prevention in children [29]. We made the following modifications; 1) We excluded the symptom of “chest tightness” because young children are not able to express this symptom objectively [30,31], 2) we also excluded measurement of peak expiratory flow and/or spirometry because children less than five years are not able to perform these tests effectively [32]. Lastly, we included chest x-rays to aid the distinction between asthma and pneumonia. Pneumonia is very common in Uganda and among children less than five years, the clinical presentation of asthma and pneumonia are very similar [1,33].

***Bronchiolitis*** was defined based on South African guidelines for diagnosis, management and prevention of acute viral bronchiolitis [34]. It was defined as an acute illness in children less 2 years of age characterized by mild upper respiratory tract signs, low-grade fever, hyper-inflation of the chest and wheezing. Severe cases present with tachypnoea and lower chest wall retractions.

***Pneumonia:*** We defined pneumonia as presence of cough and/or difficult breathing, and fast breathing with/without chest retractions [2]. However, studies among children with WHO-defined pneumonia have indicated that using this approach, pneumonia, and particularly bacterial pneumonia is over-diagnosed [35,36]. Therefore the following modifications were made to improve on the specificity of this definition.

- We included results of chest x-ray to help distinguish viral and bacterial pneumonia in some children. We acknowledge that chest x-ray findings alone cannot be used to differentiate viral from bacterial pneumonia [37,38]. However, in some cases, the chest x-ray findings imply a particular aetiology. For example, consolidation is associated with streptococcus pneumoniae infection whereas pneumatoceles are characteristic of Staphylococcal infection [37,39].
- We also included test results for white cell count (total and differential), blood culture and serum C-reactive protein, to help differentiate viral from bacterial pneumonia. Again, we acknowledge that these tests do not expressly identify the aetiology of pneumonia [40]. However, when used in combination with history and examination findings, they can be helpful in identifying the cause of the pneumonia.
- We included fever to help us distinguish pneumonia and asthma syndrome. Fever was defined as caretaker’s report of the child being hot and/or axillary temperature of ≥38°C. Fever is more likely to be present in children with pneumonia compared to those with asthma syndrome [28].

The term “Asthma syndrome” was used to refer to children with acute asthma and bronchiolitis because in young children, it is difficult to differentiate acute asthma from bronchiolitis due to the overlap in clinical presentation [1]. Tachypnoea was defined according to WHO guidelines on pneumonia [2]. Normalization of respiratory rate was defined as having a rate of less than 50 breaths per minute for infants, and below 40 breaths per minute for children above one year, on two consecutive readings 12 hours apart. Normalization of oxygen saturation (SaO_2_) was defined as SaO_2_ ≥ 92% while breathing room air for at least 15 minutes, and maintaining the same on at least two consecutive readings, 12 hours apart. Prolonged hospitalization was regarded as having stayed in hospital for a period greater than 4 days [18].

### Statistical analysis

The sample size was calculated based on a study in India that looked at factors associated with prolonged hospitalization among children admitted with WHO-defined severe pneumonia [41]. To determine the proportion of children with prolonged duration of hospitalization, a minimum sample size of 373 children was calculated, at 95% confidence level and power of 80%. Allowing for 10% attrition, the minimum total sample size was 411 children. However, the current paper was part of a bigger study involving 614 children and all were included in the analysis.

Data was double-entered in Epidata version 3.0 and exported to Stata version 12.0 (Stata Corp, College station USA) for analysis. To determine factors independently associated with prolonged hospitalization and mortality among children with asthma syndrome and pneumonia, multivariable analysis was done. A Cox regression model was built by including all factors with a p value ≤0.2 at bivariate analysis. Multicolinearlity and interaction of the predictor variables was checked until we obtained the best fitting model. Hazard and Risk ratios at 95% confidence intervals (CIs) were derived. A log rank test was used to compare duration of hospitalization among children with asthma syndrome and pneumonia. A p value of ≤0.05 was considered statistically significant. Children who died and those who were lost to follow up were censored.

## Results

### General overview

**Figure 1:**
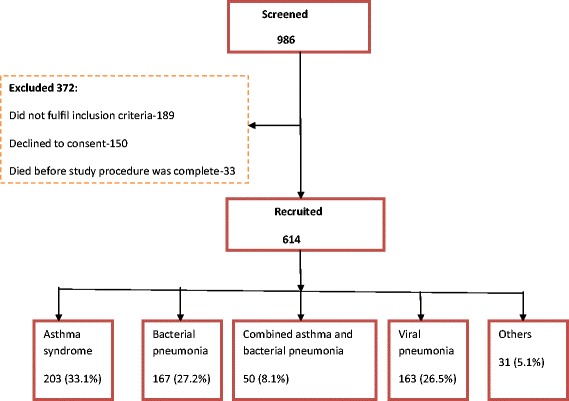
**Study profile.**

From August 2011 to June 2012, nine hundred and eighty six (986) children aged 2 to 59 months who presented with cough and/or difficult breathing were screened. Of these 614 were recruited. The remaining 372 children were not recruited because: 189 (50.8%) did not fulfill the inclusion criteria, 150 (40.3%) had caretakers who declined to participate and 33 (8.9%) died before any investigations could be done (Figure 1). The median age was 10 months (inter-quartile range 6–18 months) and 333 (54.2%) were less than 12 months old. Of the 614 participants, 592 (96.4%) children were followed up till discharge or death while 22 (3.6%) were lost to follow up. Of the 592 children who were followed up, 35 (5.9%) were managed as out-patients and 5 (0.8%) died within the first 12 hours. Five hundred and ninety three (96.6%) of children had chest in-drawing and hence fulfilled the WHO definition of severe pneumonia.

The expert review panel made *post hoc* diagnoses and found that 253 (41.2%) of the participants had asthma syndrome, 167 (27.2%) had bacterial pneumonia, 163 (26.5%) had viral pneumonia, and 31 (5.1%) had other diagnoses like pulmonary tuberculosis. Of the children with asthma syndrome, 50 (19.8%) had combined asthma and bacterial pneumonia. The rest of the characteristics are summarized in Table 1.

**Table 1:** **Baseline characteristics of the study participants (N = 614)**

**Variable**	**Number**	**Percentage (%)**
**Sex**		
Male	347	56.5
**Age**		
<12 months	333	54.2
**Nutrition status**		
Normal	419	68.2
Stunting (HAZ score < −2SD)	124	20.1
Wasting (WHZ score < −2SD)	51	8.3
Anthropometry not done	19	3.1
**HIV infection**		
Positive	41	6.7
Negative	548	89.2
HIV test not done	25	4.1
**Residence**		
Rural	146	23.8
Urban	468	76.2

### Outcome measures

#### a) Mortality

**Figure 2:**
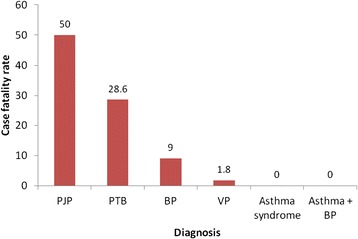
**Case fatality among ‘under-fives’ with acute respiratory symptoms in Mulago hospital Uganda.**

Overall mortality was 3.6% (22/614). The average duration of hospital stay before death was 3 days. Six (27.3%) of the 22 deaths occurred within 24 hours of admission. The majority (63.6%) of deaths were among children less than 12 months. Pneumonia alone contributed 20 (90.9%) of the total deaths. The highest case fatality was among children with *pneumocystis jirovecii* pneumonia (50%), and pulmonary tuberculosis (28.6%) while that for bacterial pneumonia was 9.0% and viral pneumonia was 1.2%. None of the children with asthma syndrome alone (asthma + bronchiolitis) or combined asthma and bacterial pneumonia died (Figure 2). Of the 203 children with asthma syndrome, 33 (16.3%) did not have a prescription for bronchodilators. One hundred and ninety eight (198) of the 203 children with asthma syndrome had chest indrawing and hence needed systemic steroids. However, 68.2% (135/198) of them did not have a prescription for the steroids.

#### Factors associated with mortality among the participants

Children with hypoxemia (SaO_2_ < 92%) at admission were more likely to die (HR = 10.7, 95% CI 1.4 - 81.1) compared to those with normal peripheral oxygen saturation. Similarly, children with severe acute malnutrition were more likely to die (HR = 5.7, 95% CI 2.1 - 15.8) compared to those with normal nutritional status and underweight. Age, gender, lack of exclusive breastfeeding for at least 3 months, and presence of co-morbidities like malaria and HIV infection were not associated with mortality (Table 2).

**Table 2:** **Factors associated with mortality among children aged 2–59 months admitted with acute respiratory symptoms in Mulago hospital Uganda**

**Variable**	**HR (Unadjusted) 95% CI**	**HR (Adjusted) 95% CI**	**P value**
Age <12 months	1.3 (0.5 – 3.4)	-	-
Oxygen saturation (SaO_2_) < 92%	12.2 (1.6 – 92.0)	10.7 (1.4 – 81.1)	0.022
Axillary temperature ≥38°C	2.1 (0.6 – 7.5)	-	-
HIV infection	1.9 (0.5 – 6.8)	-	-
Malaria	1.2 (0.4 – 3.2)	-	-
Severe acute malnutrition (WHZ <2SD)	6.4 (2.3 – 17.9)	5.7(2.1 – 15.8)	0.001
Lack of exclusive breastfeeding for at least 3 months	1.7 (0.6 – 4.5)	-	-
Low level of education of caretaker	3.5 (0.9 – 13.3)	-	-
Male sex	2.1 (0.8 – 2.2)	-	-

HR- Hazard ratio, CI-Confidence interval.

#### b) Duration of hospitalization for children with asthma syndrome and pneumonia

The average duration of hospital stay was 4.0 days (SD 4.3 days). Twenty seven (4.4%) of the total participants were admitted to the intensive care unit. Complications including pleural effusion, empyema thoracis and septicaemia were noted in 18 (2.9%) of the children.

**Figure 3:**
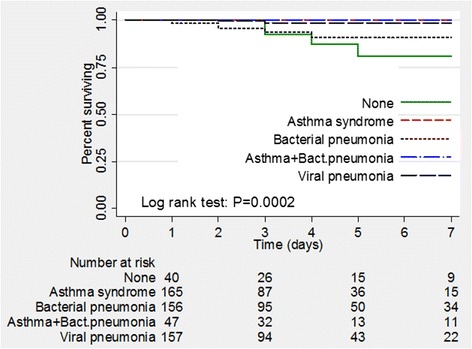
**Kaplan Meier curves comparing duration of hospitalization among the diagnoses.**

**Figure 4:**
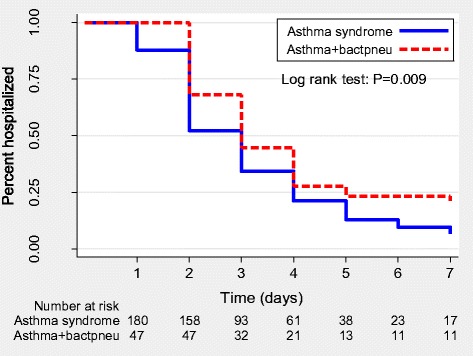
**Kaplan Meier curves comparing duration of hospitalization of children with combined asthma and bacterial pneumonia and asthma syndrome alone.**

We compared the duration of hospitalization among children with asthma syndrome (acute asthma + bronchiolitis), bacterial pneumonia, combined asthma and bacterial pneumonia and, viral pneumonia. We found a statistically significant difference in duration of hospitalization of the children diagnosed with either of the above conditions (p < 0.001, log rank test- Figure 3). Children with asthma syndrome had the shortest duration of hospitalization while those with bacterial pneumonia had the longest duration of hospital stay. Children with asthma syndrome had a significantly shorter hospital stay compared to those with viral and bacterial pneumonia combined (p<0.001, log rank test). Among children with pneumonia, those who had bacterial pneumonia were more likely to be hospitalized for a longer duration compared to those with viral pneumonia (p = 0.006, log rank test). Furthermore, children who had combined asthma and bacterial pneumonia were hospitalized for a longer duration (p<0.001, log rank test) compared to those who had asthma syndrome alone (Figure 4).

#### Factors associated with prolonged hospital stay among the study participants

Severe acute malnutrition, hypoxemia (SaO_2_ < 92%), and age <12 months old were variables significantly associated with prolonged hospitalization. The factors that were significantly associated with prolonged hospitalization among the study participants with respect to asthma syndrome and pneumonia are summarised in Table 3.

**Table 3:** **Factors associated with prolonged hospital stay (>4 days) among ‘under-fives’ admitted with acute respiratory symptoms in Mulago hospital Uganda**

**Variable**	**Asthma syndrome**		**Pneumonia**	
	**RR (95% CI)**	**P value**	**RR (95% CI)**	**P value**
Age <12 months	1.2 (1.0-1.4)	0.022	1.2 (1.1 – 1.4)	0.010
SaO2 < 92%	1.4(1.2-1.7)	0.000	1.4 (1.2 – 1.6)	0.000
Male sex	1.1 (1.0-1.3)	0.134	1.1 (0.8 – 1.4)	0.542
Severe acute malnutrition	1.5(1.3-1.8)	0.000	3.1 (2.3 – 4.1)	0.013
Low level of education of caretaker	1.0(0.7-1.4)	0.926	1.2 (0.7 – 2.2)	0.463
Lack of exclusive breastfeeding for at least 3 months	1.1 (0.9-1.2)	0.300	1.3 (1.0 – 1.7)	0.056
Prematurity	1.0(0.7-1.4)	0.929	1.3 (0.7 – 2.2)	0.395
RSV infection	1.2 (1.0-1.4)	0.068	1.2 (0.9 – 1.7)	0.217
Malaria	1.0(0.9-1.2)	0.715	1.2 (0.9 – 1.6)	0.310

RR- Risk ratio, CI-Confidence interval.

#### c) Time to normalization of oxygen saturation and respiratory rate

The mean time to normalization of peripheral oxygen saturation was 1.3 days for children with asthma syndrome and 1.6 days among those with pneumonia and the difference was not statistically significant (p = 0.097 log rank test). Normalization of respiratory rate was significantly faster among children with asthma syndrome compared to those with pneumonia (p < 0.05, log rank test).

#### Co-morbidities

Malaria, HIV infection and severe acute malnutrition were the major co-morbidities among the participants. Overall, 162 (26.4%) had malaria and 41 of 589 (7.0%) had HIV infection. Severe acute malnutrition was noted in 51 of 595 (8.6%) of the children. Malaria was most common among children with viral pneumonia (31.5%) while severe acute malnutrition was most common among children with bacterial pneumonia (Table 4).

**Table 4:** **Co-morbidities among young children with asthma syndrome and pneumonia in Mulago hospital Uganda**

**Variable**	**Asthma syndrome (N=203)**	**Bacterial pneumonia (N = 167)**	**Combined asthma and bacterial pneumonia (N = 50)**	**Viral pneumonia (N = 163)**
	**n(%)**	**n (%)**	**n (%)**	**n (%)**
Malaria	52 (25.6)	39 (23.4)	9 (18.0)	51 (31.5)
HIV infection	3 (1.5)	15 (9.4)	3 (6.4)	13 (8.4)
Severe acute malnutrition	3 (1.5)	25 (15.8)	2 (4.2)	11 (6.8)
Under-weight	29 (14.3)	39 (24.7)	11 (22.9)	40 (24.8)

## Discussion

In this study, we describe the immediate clinical outcomes of children aged 2–59 months who were admitted with acute respiratory symptoms in Mulago hospital Uganda. We compared the outcomes of children with asthma syndrome and pneumonia with specific reference to mortality, duration of hospitalization and resolution of signs.

### Mortality

Overall mortality was 3.6%. Of the 22 children who died, 20 (90.9%) were diagnosed with pneumonia. None of the children with asthma syndrome died. The current low mortality of 3.6% indicates a sharp decline when compared to previous studies [16-18] on children less than five years, with WHO-severe pneumonia in Mulago hospital in which the mortality was very high ranging from 11.9% to 24%. The reasons for the low mortality in this study are not very clear. However, it is possible that the focus on diagnosing and treating of most children who presented with wheezing may have played a role. According to the findings in a recent study by our team, on the same population, many children with asthma syndrome were misdiagnosed as pneumonia by the ward doctors [6]. Indeed, when the ward case files of the children in this study were reviewed, we found that 16.3% and 68.2% of the children with asthma syndrome had no prescriptions for bronchodilators and steroids respectively. Yet, untreated asthma is associated with mortality [11]. It is possible that some of the case fatalities in the previous studies were due to undiagnosed asthma. In this study, we ensured that all children with wheezing got appropriate doses of bronchodilators and systemic steroids. Optimally treated asthma is generally associated with low mortality [42]. Hence, having ensured that all children with asthma syndrome received optimal treatment may explain the overall low mortality in this study.

### Duration of hospitalization

The average duration of hospitalization was significantly shorter among children with asthma syndrome (2 days) compared to those with pneumonia (4 days). In addition, the children with asthma syndrome had quicker normalization of respiratory rate and oxygen saturation. Similar studies have also indicated that children less than five years admitted with asthma generally have short hospital stay [42,43]. This may be explained by the underlying pathophysiological mechanisms in acute asthma; smooth muscle contraction and bronchiolar inflammation with little or no involvement of lung parenchyma, which once treated appropriately, hastens resolution of symptoms.

### Factors associated with prolonged hospitalization among children with asthma syndrome and pneumonia

Infants and children with hypoxemia and severe acute malnutrition had prolonged hospitalization regardless of their primary diagnosis (asthma syndrome or pneumonia). Infants tend to present with more severe disease and usually have longer duration of hospitalisation because of their immunity which is not yet well developed to fight diseases effectively [41]. Similarly, children with severe malnutrition tend to have longer course of illness due to low immunity [44]. Children with hypoxemia also experienced longer duration of hospitalization. Similar findings have been revealed by previous researchers [10,42]. Hypoxemia is a measure of disease severity; hence, children with more severe signs and symptoms tend to be hospitalized longer compare to those with milder disease.

### Methodological considerations

To our knowledge, this is among the few studies in Africa, and Uganda in particular, that have described the immediate clinical outcome of children with acute asthma and also made comparisons with pneumonia. The study used a prospective design as opposed to earlier studies describing asthma outcomes that were retrospective [12,14,42]. In addition, the patients were monitored every 12 hours for key clinical signs such as respiratory rate and oxygen saturation. This makes the findings fairly accurate and hence can be generalizable to hospital settings in Uganda which are similar to Mulago hospital.

This was purely an observation study. The research team did not play an active role in management of the patients during follow up other than relaying test results and any other relevant information on status of the patients to the ward doctors. In addition, the decision to discharge was at the discretion of the ward doctors. This may have influenced the duration of hospitalization among some of the study participants. However, when we compared the time to resolution of respiratory rate and oxygen saturation (which are objective measures of disease severity) among children with asthma syndrome and pneumonia, we found that children with asthma syndrome took a shorter duration to normalization of these parameters. Therefore, the findings that children with asthma syndrome have a shorter course of illness than those with pneumonia were deemed true.

Although standard protocols for management of children with pneumonia are available in the hospital, the study team had no role in ensuring that they are strictly followed. Choice of antibiotics was made by the attending doctors. Furthermore, we were not able to ensure that every participant gets zinc, which is known to influence mortality among children with severe pneumonia, particularly those with HIV infection [18]. Such differences in management approaches may have influenced outcomes in some children.

We were unable to study some factors such as serum electrolytes, blood sugar, which may influence outcomes of children admitted with acute respiratory illnesses.

The primary outcome of this study was duration of hospitalization and not mortality. Therefore, the sample size may not have been adequate to reveal all the factors associated with mortality. Furthermore, this being the first study in Uganda to look at outcomes of children with acute asthma, we were unable to document the effect of untreated asthma on mortality. Further research specifically looking at mortality among children with acute asthma and pneumonia is recommended. Indeed, a randomized controlled trial (No. NCT01868113) looking at treatment of children with acute respiratory infections and asthma among under-fives in Mulago hospital, with respect to morbidity and mortality is on-going.

## Conclusions

This study identified a sharp decline in acute respiratory mortality compared to the previous studies in Mulago hospital. This may partly be related to focus on and treatment of acute asthma in this study, and will be analysed in a later study. Pneumonia still causes significant mortality among children less than five years. Hypoxemia, severe acute malnutrition, and being an infant were associated with poor prognosis among young children with asthma syndrome and pneumonia. Such factors need to be considered when designing/reviewing management protocols for asthma and pneumonia in Mulago hospital. Children with asthma syndrome (acute asthma and bronchiolitis) if appropriately managed, have favourable outcomes. Furthermore, these findings reiterate the importance of nutrition as a child survival strategy for both communicable and non-communicable diseases. There is need to develop and also strengthen current strategies to avert the high morbidity and mortality associated with asthma and pneumonia among children less than five years.

## Additional file

Additional file 1:
**STROBE statement—checklist of items that were addressed in the study.**
