# Supplementary material for: Clinical outcomes of children with acute asthma and pneumonia in Mulago hospital, Uganda: a prospective study
Source: BMC Pediatr. 2014 Nov 28;14:285. doi: 10.1186/s12887-014-0285-4 (PMC4254222; doi:10.1186/s12887-014-0285-4)
Supplement: Additional file 1: — STROBE statement—checklist of items that were addressed in the study. [file 12887_2014_285_MOESM1_ESM.doc]

**STROBE Statement—Checklist of items that were addressed in the study**

|  | Item No | | Recommendation | | Comment |
| --- | --- | --- | --- | --- | --- |
| **Title and abstract** | 1 | | (*a*) Indicate the study’s design with a commonly used term in the title or the abstract | | This study was a prospective study and this has been included in the title |
|  |  |  | (*b*) Provide in the abstract an informative and balanced summary of what was done and what was found | | We have included summarized information on the methods and findings |
| Introduction | | | | |  |
| Background/rationale | 2 | | Explain the scientific background and rationale for the investigation being reported | | The scientific background on outcomes of children with asthma and pneumonia and the rationale for this study are provided in the first three paragraphs of the section on background. |
| Objectives | 3 | | State specific objectives, including any pre-specified hypotheses | | The specific objectives were;  i)To compare the immediate clinical outcomes of children diagnosed with acute asthma and pneumonia  ii) To describe the factors associated with prolonged hospitalization and mortality among the study participants. This information has been outlined in the last paragraph of the background section. |
| Methods | | | | |  |
| Study design | 4 | | Present key elements of study design early in the paper | | This was a prospective study and this has been indicated in the first sentence under the section on methods |
| Setting | 5 | | Describe the setting, locations, and relevant dates, including periods of recruitment, exposure, follow-up, and data collection | | The study setting was the emergency and general paediatric wards of Mulago hospital. The setting has been described under the first section on methods (study design and setting). |
| Participants | 6 | | (*a*) Give the eligibility criteria, and the sources and methods of selection of participants. Describe methods of follow-up | | The details on the participants, recruitment, measurements and follow up have been described under the sub-section of *“Recruitment, management and follow up of participants”.* All these aspects have been presented under the same sub-heading to maintain logical flow of the processes that were followed. |
|  |  |  | (*b*) For matched studies, give matching criteria and number of exposed and unexposed | | Not applicable. This was a prospective unmatched study. |
| Variables | 7 | | Clearly define all outcomes, exposures, predictors, potential confounders, and effect modifiers. Give diagnostic criteria, if applicable | | Under the sub-section of *‘Variables’* and *Definitions’,* the details of the outcomes are provided. These were; duration of hospitalization, time to normalization of oxygen saturation and respiratory rate, and mortality. In this study, the diagnoses were made *post hoc* and the diagnostic criteria/study definitions have been described and where necessary, the references have been provided. The predictor variables have also been provided. |
| Data sources/ measurement | 8 | | For each variable of interest, give sources of data and details of methods of assessment (measurement). Describe comparability of assessment methods if there is more than one group | | The details on how the data for the different variables was obtained and how the measurements were done have been included under the section on participants. This was done to maintain a clear and logical flow of what was done throughout the study procedure. |
| Bias | 9 | | Describe any efforts to address potential sources of bias | | The methods that were used to calculate the sample size are provided under the section on statistical analysis  Multivariable and survival analysis were the methods used for data analysis. In order to control for confounding, a Cox regression model was built using manual backward model selection.  The details of the statistical methods and they were applied are provided under the section on statistical analysis |
| Study size | 10 | | Explain how the study size was arrived at | |  |
| Quantitative variables | 11 | | Explain how quantitative variables were handled in the analyses. If applicable, describe which groupings were chosen and why | |  |
| Statistical methods | 12 | | (*a*) Describe all statistical methods, including those used to control for confounding | |  |
|  |  |  | (*b*) Describe any methods used to examine subgroups and interactions | |  |
|  |  |  | (*c*) Explain how missing data were addressed | | There was no missing data on the outcomes of interest |
|  |  |  | (*d*) If applicable, explain how loss to follow-up was addressed | | The children who were lost to follow up were censored and this has been indicated in the section on statistical analysis |
|  |  |  | (*e*) Describe any sensitivity analyses | | Not applicable |
| Results | | | | |  |
| Participants | | 13 | | (a) Report numbers of individuals at each stage of study—eg numbers potentially eligible, examined for eligibility, confirmed eligible, included in the study, completing follow-up, and analysed | A flow chart (Figure 1) has been included to clearly show the number that was screened and those who were recruited |
|  |  |  |  | (b) Give reasons for non-participation at each stage |  |
|  |  |  |  | (c) Consider use of a flow diagram |  |
| Descriptive data | | 14 | | (a) Give characteristics of study participants (eg demographic, clinical, social) and information on exposures and potential confounders | The characteristics of the study participants have been outlined in table 1.  The details on follow up time before discharge is described under the section on *‘Duration of hospitalization for children with asthma syndrome and pneumonia’* while the follow up time before death is described under the section on ‘*Mortality’.*  There was no missing data on the variables of interest |
|  |  |  |  | (b) Indicate number of participants with missing data for each variable of interest |  |
|  |  |  |  | (c) Summarise follow-up time (eg, average and total amount) |  |
| Outcome data | | 15 | | Report numbers of outcome events or summary measures over time | The unadjusted and adjusted hazard ratios and p values for factors associated with mortality are provided in table 2.  The adjusted risk ratios for factors associated with prolonged hospitalization are outlined in table 3. |
| Main results | | 16 | | (*a*) Give unadjusted estimates and, if applicable, confounder-adjusted estimates and their precision (eg, 95% confidence interval). Make clear which confounders were adjusted for and why they were included |  |
|  |  |  |  | (*b*) Report category boundaries when continuous variables were categorized | The continuous variables in this study were; temperature, peripheral oxygen saturation, age, and respiratory rate as described in the section on methods. The categorization of these variables is indicated in table 2 and 3. |
|  |  |  |  | (*c*) If relevant, consider translating estimates of relative risk into absolute risk for a meaningful time period | In this study, the major interest was to compare the risk of a given outcome, say prolonged hospitalization between children with asthma syndrome and pneumonia. Hence, the most relevant measurement was relative risk rather than absolute risk |
| Other analyses | | 17 | | Report other analyses done—eg analyses of subgroups and interactions, and sensitivity analyses | Not applicable |
| Discussion | | | | |  |
| Key results | | 18 | | Summarise key results with reference to study objectives | The results were discussed with respect to the objectives which have been presented as sub-headings of ‘*Mortality’, Duration of hospitalization’* and *‘Factors associated with prolonged hospitalization’* |
| Limitations | | 19 | | Discuss limitations of the study, taking into account sources of potential bias or imprecision. Discuss both direction and magnitude of any potential bias | The study limitations are discussed under the section on ‘*Methodological considerations’.*  In addition, interpretation, strengths and weaknesses of the individual findings with respect to available literature have been discussed under each objective. |
| Interpretation | | 20 | | Give a cautious overall interpretation of results considering objectives, limitations, multiplicity of analyses, results from similar studies, and other relevant evidence |  |
| Generalisability | | 21 | | Discuss the generalisability (external validity) of the study results | The generalizability of the results in this study is limited to settings similar to Mulago hospital. This fact has been alluded to under methodological considerations. |
| Other information | | | | |  |
| Funding | | 22 | | Give the source of funding and the role of the funders for the present study and, if applicable, for the original study on which the present article is based | The study was funded by Danish Ministry of Foreign Affairs (DANIDA) and this information has been included in the section on acknowledgement |
